# Austin District Med. Society, Annual Meeting

**Published:** 1894-01

**Authors:** 


**The Austin District Medical Society** held its regular annual meeting in Austin, December 21, ult., and it was an interesting occasion. The retiring President, Dr. R. P. Talley, of Temple, delivered an address characteristic of the man; he reversed the order of glorifying medicine and medical men, of lauding "our noble profession," and showed up some practices and things of which we have not much right to be very proud. He was severe on a certain class of specialists. The address will appear in our next issue. Dr. T. J. Bennett, of Austin, the founder of the Society and long-time Secretary, was honored by election to the Presidency, and Dr. S. E. Hudson, of the JOURNAL, was elected Secretary. Dr. M. M. Smith read a paper on Ostitis, and a paper by Dr. Milliken, of New York, on Varicocele was read. [Published herewith.] Dr. J. C. Anderson, of Granger, related the clinical history of an unusual case in surgical practice, and will, by request of the Society, report it in writing at next meeting, for publication. Dr. Barnwell, who sewed up the laceration in the little Bohemian girl, the victim of the brute Nichols, who is to be executed at Austin on 12th inst. (having been respited from Dec. 22d ult.), will, by request, submit a report of the case; the fact of the girl's recovery making the case one of very great interest. Dr. W. J. Mathews exhibited a patient who had recovered from a large abscess of the liver, and gave the history of the case; evacuated a gallon of pus.

The subject "Shall Consumption be Quarantined?" was announced on the program, but was not discussed very extensively, as there appeared to be some misunderstanding of what was intended. The subject was not fairly stated. No one proposes to quarantine consumption in the sense of isolation and detention; but it is a very proper subject for discussion whether consumptives should be permitted to travel in the same coach or boat or stage with other passengers, and whether or not hotel keepers or palace car men should not be required to enforce sanitary measures after each occupancy of car or room by a consumptive. The subject will come up again in a different shape next meeting.

A splendid banquet at night closed the annual exercises, and was much enjoyed. The Society is still growing.

